# An RNAi Screen To Identify Protein Phosphatases That Function Within the *Drosophila* Circadian Clock

**DOI:** 10.1534/g3.116.035345

**Published:** 2016-10-25

**Authors:** Parul Agrawal, Paul E. Hardin

**Affiliations:** * Department of Biology, Texas A&M University, College Station, Texas 77845-3258; † Center for Biological Clocks Research, Texas A&M University, College Station, Texas 77845-3258

**Keywords:** *Drosophila melanogaster*, activity rhythms, protein phosphatases, circadian clocks

## Abstract

Circadian clocks in eukaryotes keep time via cell-autonomous transcriptional feedback loops. A well-characterized example of such a transcriptional feedback loop is in *Drosophila*, where CLOCK-CYCLE (CLK-CYC) complexes activate transcription of *period* (*per*) and *timeless* (*tim*) genes, rising levels of PER-TIM complexes feed-back to repress CLK-CYC activity, and degradation of PER and TIM permits the next cycle of CLK-CYC transcription. The timing of CLK-CYC activation and PER-TIM repression is regulated posttranslationally, in part through rhythmic phosphorylation of CLK, PER, and TIM. Previous behavioral screens identified several kinases that control CLK, PER, and TIM levels, subcellular localization, and/or activity, but two phosphatases that function within the clock were identified through the analysis of candidate genes from other pathways or model systems. To identify phosphatases that play a role in the clock, we screened clock cell-specific RNA interference (RNAi) knockdowns of all annotated protein phosphatases and protein phosphatase regulators in *Drosophila* for altered activity rhythms. This screen identified 19 protein phosphatases that lengthened or shortened the circadian period by ≥1 hr (*p* ≤ 0.05 compared to controls) or were arrhythmic. Additional RNAi lines, transposon inserts, overexpression, and loss-of-function mutants were tested to independently confirm these RNAi phenotypes. Based on genetic validation and molecular analysis, 15 viable protein phosphatases remain for future studies. These candidates are expected to reveal novel features of the circadian timekeeping mechanism in *Drosophila* that are likely to be conserved in all animals including humans.

A diverse array of organisms use circadian (∼24 hr) clocks to regulate daily rhythms in physiology, metabolism, and behavior. These clocks keep time via self-sustained transcriptional feedback loops that are synchronized to daily environmental cycles and drive daily rhythms in gene expression. As in other animals, the *Drosophila* core timekeeping loop is activated by two bHLH‐PAS transcription factors, CLOCK and CYCLE (CLK-CYC), and repressed by PERIOD-TIMELESS (PER-TIM) complexes. The generation of self-sustaining oscillations depends on posttranslational regulation of clock proteins, which modulates their stability, activity, and/or subcellular localization during the daily cycle. Multiple levels of posttranslational controls are built into this oscillatory system to produce a ∼24 hr period, support a robust cycling amplitude, and enable resetting by environmental inputs.

The best characterized posttranslational modification of clock proteins is phosphorylation. In *Drosophila*, nuclear localization of the PER-TIM repressor complex is regulated by CASEIN KINASE II (CK2) and SHAGGY (SGG) phosphorylation (Martinek *et al.* 2001; Lin *et al.* 2002; Akten *et al.* 2003, 2009), whereas PER degradation in the nucleus is regulated by DOUBLE-TIME (DBT) and NEMO (Kloss *et al.* 1998, 2001; Price *et al.* 1998; Chiu *et al.* 2008, 2011). Phosphorylation also controls CLK-CYC function where NEMO, DBT, and CK2-dependent phosphorylation control CLK stability and activity (Kim and Edery 2006; Yu *et al.* 2006; Szabo *et al.* 2013). The phosphorylation state of a protein is controlled dynamically by protein kinases and phosphatases. However, few phosphatases have been identified that function in the *Drosophila* circadian clock; Protein Phosphatase 2a (PP2a) and Protein Phosphatase 1 (PP1), control PER-TIM repressor stability and nuclear localization (Sathyanarayanan *et al.* 2004; Fang *et al.* 2007), and the PP2a-STRIPAK complex dephosphorylates CLK to promote CLK-CYC transcription (Andreazza *et al.* 2015). Despite our understanding of how phosphorylation controls nuclear localization and degradation of PER-TIM repressor complexes and activity of CLK-CYC activator complexes, the role phosphorylation plays in controlling cytoplasmic PER-TIM accumulation and the light-dependent TIM degradation are not well understood. These events control progression through the feedback loop, and are therefore critical for controlling the period, phase, and amplitude of rhythmic transcription.

To determine how dephosphorylation regulates rhythmic transcription within the *Drosophila* clock, we used RNAi knockdown to screen all annotated protein phosphatases in *Drosophila* for defects in locomotor activity rhythms. Of 86 protein phosphatase or protein phosphatase regulator genes screened, 19 showing period alterations or arrhythmicity were identified as candidate clock protein phosphatases, including LEUKOCYTE ANTIGEN-LIKE (LAR), which is required to build a neuronal circuit in the *Drosophila* brain that mediates circadian activity rhythms (Agrawal and Hardin 2016). Additional genetic reagents were obtained or generated to validate the RNAi phenotypes. The validated phosphatases identified here may contribute to clock cell development or the circadian timekeeping mechanism, and represent potential genetic links to clock-associated disorders in humans and novel targets for the development of drugs to treat such disorders.

## Materials and Methods

### Fly stocks

The *w*^1118^ and *w*^1118^;*CyO*/*Sco*;TM2/TM6B strains were used as wild-type controls for activity rhythms and as balancers to generate lines used for screening and analysis, respectively. The following Gal4 strains were used to drive RNAi expression in clock cells: *w*^1118^;*tim*Gal4^62^, *w*^1118^;;*pdf*Gal4, and *w*^1118^;;*tim*Gal4^16^. The following RNAi strains from the Vienna *Drosophila* RNAi Center (VDRC) were used to knockdown phosphatase/regulator expression in clock cells, listed as the gene name and abbreviation or CG number followed by the VDRC line number in parenthesis: *puckered*, *puc* (GD3018); *Protein tyrosine phosphatase 52F*, *Ptp52F* (GD3116); *TbCMF46* (GD17123); *string*, *stg* (GD17760); CG17124 (GD19078); *Calcineurin B*, *CanB* (GD21611); *Protein phosphatase 19C*, *Pp4-19C* (GD25317); *Protein tyrosine phosphatase 4E*, *Ptp4E* (GD27232); *Calcineurin A1*, *CanA1* (GD32283); CG17598 (GD32956); *Protein phosphatase 1 at 87B*, *Pp1-87B* (GD35025); *Phosphotyrosyl phosphatase activator*, *Ptpa* (GD41912); *microtubule star*, *mts* (GD41924); *sds22* (GD42051); *Mitogen-activated protein kinase phosphatase 3*, *Mkp3* (GD45415); *Protein phosphatase 2A at 29B*, *Pp2a-29B* (GD49671); CG6380 (KK100121); CG17746 (KK100178); CG2104 (KK100216); *Mapmodulin* (KK100283); *Glycogen binding subunit 70E*, *Gbs-70E* (KK100593); CG42327 (KK100914); *Ppm1* (KK101257); CG10376 (KK101335); *widerborst*, *wdb* (KK101406); *Calcium and integrin binding family member 2*, *Clb2* (KK101474); *Inhibitor-2*, *I-2* (KK101547); *Protein phosphatase V*, *PpV* (KK101997); *Protein phosphatase Y at 55A*, *PpY-55A* (KK102021); *Protein phosphatase N at 58A*, *PpN-58A* (KK102060); CG14297 (KK102071); *Protein tyrosine phosphatase 36E*, *Ptp36E* (KK102397); CG31469 (KK102474); *Glycogen binding subunit 76A*, *Gbs-76A* (KK103044); *Protein phosphatase 2B at 14D*, *Pp2B-14D* (KK103144); CG32568 (KK103317); CG7115 (KK103354); *Cep97* (KK103357); *cell division cycle 14*, *cdc14* (KK103627); *Protein tyrosine phosphatase Meg*, *Ptpmeg* (KK103740); CG32812 (KK104081); *twins*, *tws* (KK104167); *Protein phosphatase D6*, *PpD6* (KK104211); *MAP kinase-specific phosphatase*, *Mkp* (KK104374); *Protein tyrosine phosphatase Meg2*, *Ptpmeg2* (KK104427); *Protein phosphatase D5*, *PpD5* (KK104452); *flapwing*, *flw* (KK104677); CG11597 (KK104729); *Protein tyrosine phosphatase 69D*, *Ptp69D* (KK104761); *Protein Tyrosine Phosphatase mitochondrial 1*, *PTPMT1* (KK104774); *Dullard*, *Dd* (KK104785); *myopic*, *mop* (KK104860); *MAPK Phosphatase 4*, *MKP-4* (KK104884); CG13197 (KK105122); *Protein phosphatase 2C*, *Pp2C* (KK105249); *Protein phosphatase 4 regulatory subunit 2-related protein*, *PPP4R2r* (KK105399); *alphabet*, *alph* (KK105483); CG15528 (KK105484); *Protein phosphatase 1*α *at 96A*, *Pp1*α*-96A* (KK105525); *inhibitor-t*, *I-t* (KK105565); CG6036 (KK105568); CG5026 (KK105674); CG7378 (KK106098); CG10417 (KK106180); *TFIIF-interacting CTD phosphatase*, *Fcp1* (KK106253); *PP2A-B*’ (KK107057); *Protein phosphatase D3*, *PpD3* (KK107386); CG4733 (KK107621); *Protein phosphatase 1 at ^13^C*, *Pp1-^13^C* (KK107770); *Leukocyte-antigen-related-like*, *Lar* (KK107996); *slingshot*, *ssh* (KK107998); *eyes absent*, *eya* (KK108071); *corkscrew*, *csw* (KK108352); *Protein tyrosine phosphatase 99A*, *Ptp99A* (KK108505); CG10089 (KK108744); CG8509 (KK108802); *Nuclear inhibitor of Protein phosphatase 1*, *NIPP1* (KK108859); *Protein tyrosine phosphatase 61F*, *Ptp61F* (KK108888); *Protein phosphatase 1*, *Y-linked 2*, *PP1-Y2* (KK109147); CG14411 (KK109622); *Calcineurin A at 14F*, *CanA-14F* (KK109858); CG3632 (KK110167); *PH domain leucine-rich repeat protein phosphatase*, *Phlpp* (KK110360); *Protein tyrosine phosphatase 10D*, *Ptp10D* (KK110443); *IA-2 protein tyrosine phosphatase*, *IA-2* (KK110595); and CG3530 (KK110786). The following strains were used to characterize candidate clock protein phosphatases: UAS-*mts*RNAi (GD35171), *w*^1118^;;UAS-*mts*, *w*^1118^;P{EP}*Pp2A-29B*^EP2332^, *w^1118^*;P{RS3}*Pp2A-29B*^CB-5426-3^, *w*^1118^;PBac{WH}CG17746^f05041^, *y*^1^w*;P{EP}CG17746^G4827^, *y*^1^*w^1118^*;PBac{3HPy^+^}*I-2*^C362^, w*;P{UAS-*I*-*2*.HA}G/+;P{UAS-*Pp1-87B*.HA}^1^H, *y*^1^*w*^67c23^;P{SUPor-P}CG7115^KG02655^, *w*^1118^;UAS-*Cep97*, UAS-*Cep97*/Y, *w*^1118^;;UAS-*Cep97*, *y*^1^;P{SUPor-P}tocKG08989*PpD6*^KG08989^, *y*^1^w*Mi{MIC}*Ptpmeg2*^MI03011^/Y, *w*^67c23^P{lacW}*Ptpmeg2*^G0232^/Y, *y*^1^*w*^67c23^P{Mae-UAS.6.11}*Ptpmeg2*^GG01129^/Y, *w*^1118^PBac{WH}*Ptpmeg2*^f06600^, w*;;*Ptp69D*^1^, w*;;Df(3L)8ex25, *w*^1118^;;*Ptp69D*^10^, w^1118^;;*Ptp69D*^18^, *w*^1118^;;*Ptp69D*^20^, *w*^1118^;;*Ptp69D*^21^, *w*^1118^;;UAS-*Ptp69D*, *w*^1118^;;UAS-DN*Ptp69D*, *y*^1^P{SUPor-P}*MKP-4*^KG03420^, *w*^1118^;;P{GSV6}*Pp1*α*-96A*^GS11179^, *w*^1118^;;*Pp1*α*-96A*^2^, *w*^1118^;;UAS-*Pp1*α*-96A*.HA, *w*^1118^;;*Pp1*α*96A*-CRISPRmutant-1/+, *w*^1118^;;*Pp1*α*96A*-CRISPRmutant-2/+, *w*^1118^;;*Pp1*α*96A*-CRISPRmutant-3/+, *w*^1118^;UAS-*PP2C-like*, w*;*Lar*^13.2^/+, Df(2L)TW84,l(2)74i^1^,*amos*^Tft^*Lar*^TW84^/+, Df(2L)E55,rdo^1^hook^1^*Lar*^E55^pr^1^/+, *w*^1118^;;UAS-*Lar*, *w*^1118^;UAS-*CanA-14F*myc, *w*^1118^;;UAS-*CanA-14F*act-myc, *CanA-14F*-KO/Y, and UAS-CG3530RNAi (GD26216). Although *Dicer 2* enhances the transgenic RNAi effect in ∼50% of the lines tested (Dietzl *et al.* 2007), we chose not to coexpress *Dicer 2* because of the increased off-target effects and lethality that may result.

### Drosophila activity monitoring and behavior analysis

One to three d old male flies were entrained for 3 d in 12:12 light-dark (LD) and transferred to constant darkness (DD) for 7 d at 25°. The screen employed testing of each UAS-RNAi alone (as a control), driver alone (as a control), and a combination of UAS-RNAi line with *tim*Gal4 or *pdf*Gal4 (as the RNAi knockdown). Locomotor activity was monitored using the *Drosophila* Activity Monitor (DAM) system (Trikinetics). Analyses of period, power and rhythm strength during DD was carried out using ClockLab (Actimetrics) software as previously described (Pfeiffenberger *et al.* 2010). UAS-RNAi lines that produce consistent period lengthening or shortening of ≥1 hr with *p* ≤ 0.05 compared to UAS-RNAi and Gal4 driver controls or >50% arrhythmicity were analyzed further. Different genetic backgrounds may show small differences in circadian period, therefore a period change of ≤1 hr due to RNAi knockdown was not considered significant.

### Generation of Pp1α-96A CRISPR mutants

**Figure 1:**
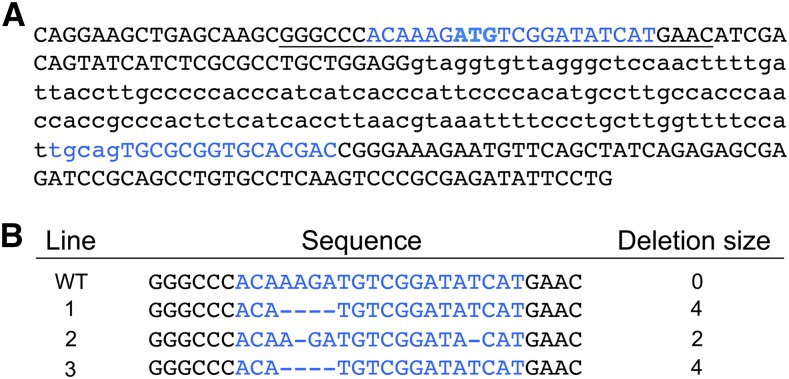
Generation of *Pp1*α*-96A* mutants using the CRISPR-Cas9 system. (A) Nucleotide sequence of the *Pp1*α*-96A* genomic region targeted for mutagenesis. Capital letters, exon sequence; lowercase letters, intron sequence; blue letters, gRNA targets; bold letters, ATG translation start codon; underlined sequence, region shown in panel (B). (B) Nucleotide sequences of control wild-type (WT) and three *Pp1*α*-96A* mutant lines. The deleted nucleotides listed are shown as dashes in the sequence. CRISPR, clustered regularly interspaced short palindromic repeats; gRNA, guide RNA.

The CRISPR-Cas9 system was used to generate deletions in *Pp1*α*-96A* (Gratz *et al.* 2015). Two guide RNAs (gRNAs) targeting the *Pp1*α*-96A* translation start and intron 1 splice acceptor sequences, 5′-ATGATATCCGACATCTTTGT-3′ and 5′-TGCAGTGCGCGGTGCACGAC-3′, were designed using the CRISPR database (http://www.flyrnai.org/crispr2/). Complementary oligonucleotides corresponding to these gRNAs were annealed and inserted into the U6-BK-gRNA vector for expression in *Drosophila* (Ren *et al.* 2013). The resulting *Pp1*α*-96A* gRNA plasmids were sequenced to confirm the integrity of the gRNA inserts (Gene Technologies Laboratory, Texas A&M University). *Pp1*α*-96A* gRNA plasmids were then sent for injection into *y*^1^ M{vas-Cas9}ZH2A *w*^1118^ embryos (Best Gene Inc.), which express *Cas9* in the germ line. Injected embryos that survived to adulthood were crossed with *w*^1118^;*CyO*/*Sco*;TM2/TM6B, and once progeny were observed, the injected adults were screened for deletions between or flanking the gRNAs. To screen for deletions, a ∼600 bp DNA fragment containing the gRNA binding sites was amplified using primers 5′-TGACCAAAGGGCGAGATTAG-3′ and 5′-ACATAGTCGCCCAGGAACAG-3′ via PCR, and sequenced. In a screen of ∼150 injected adults, three independent deletion mutants were recovered (Figure 1).

### Testing for the presence of per^SLIH^ mutation

To determine whether flies carry the *per^SLIH^* allele, a DNA fragment containing the *per^SLIH^* mutation site was amplified from genomic DNA using the Per-F (5′-GCGCTGCTCTGAATGAATCCGG-3′) and Per-R (5′-ATTGCTCACTCGTTTCCAGGACC-3′) primers via PCR. These DNA fragments were sequenced using the primer 5′- CTCCGGCAGCAGTGGCTATG-3′ to determine whether they contained a C to A nucleotide change at position 3035 corresponding to the *per*^SLIH^ mutation (Hamblen *et al.* 1998).

### Isogenizing phosphatase alleles to w^1118^

The w*;;*Ptp69D*^1^ and *CanA14F*-KO null mutants are both marked with *w^+^*, and were isogenized by backcrossing *w*^+^ male progeny to the *w^1118^* reference strain (BDSC) for seven generations.

### Data availability

The authors state that all data necessary for confirming the conclusions presented in the article are represented fully within the article.

## Results

To identify phosphatases that mediate circadian clock function, clock cell-specific RNAi knockdowns of all annotated *Drosophila* protein phosphatases were screened for altered locomotor activity rhythms. An RNAi screening strategy is advantageous because: (1) lethality can be avoided by targeting RNAi to clock cells via the Gal4/UAS system, (2) RNAi is efficient method for knocking down target gene expression, and (3) transgenic UAS-RNAi lines are available for all annotated protein phosphatase/regulator from either the VDRC, Transgenic RNAi Project at the Harvard Medical School plans (TRiP), or the National Institute of Genetics RNAi Stocks (NIG-Fly). RNAi lines from VDRC were used in this screen because the vast majority are inserted at a single genomic site (*e.g.*, KK lines) that affords efficient and comparable expression, though some lines used were inserted at random genomic sites (*e.g.*, GD lines) because KK lines for some protein phosphatases/regulators were not available. A total of 94 annotated protein phosphatase catalytic and regulatory subunits are present in the *Drosophila* genome (*Drosophila* RNAi Screening Center, DRSC). When this screen was initiated in 2010, we were able to obtain UAS-RNAi lines for 67 of these protein phosphatase catalytic and regulatory subunits and an additional 19 protein phosphatase regulators. Thus, a total of 86 protein phosphatases/regulators were screened to identify phosphatases that disrupt circadian activity rhythms.

Flies bearing UAS-RNAi and the *w*^1118^;*tim*Gal4^62^ driver or controls bearing UAS-RNAi alone and Gal4 driver alone were placed into *Drosophila* Activity Monitors, entrained for 3 d in LD cycles, and then released into DD for 7 d. Locomotor activity rhythms recorded during DD were analyzed for circadian rhythmicity and period via ClockLab software. This initial screen identified a total of 26 candidate phosphatases, all of which lengthened or shortened circadian period by ≥1 hr or were ≥50% arrhythmic (Table 1). Since many RNAi lines with a lengthened period also had a low proportion of rhythmic flies, we were concerned that the widespread RNAi knockdown of these phosphatases or ectopic RNAi expression from the *w*^1118^;*tim*Gal4^62^ driver impaired fly health. Consequently, we used *w*^1118^;;*pdf*Gal4 to restrict RNAi expression to PDF neuropeptide-expressing ventral lateral pacemaker neurons (LN_v_s) and another *tim*Gal4 insert, *w*^1118^;;*tim*Gal4^16^, to drive protein phosphatase/regulator RNAi expression. Out of the 86 lines screened with all three Gal4 drivers, 19 RNAi lines with a significantly (*p* ≤ 0.05 compared to controls) different period or >50% arrhythmic were identified (Table 1).

**Table 1:** Activity rhythms of clock cell-specific phosphatase RNAi knockdowns in *Drosophila*

Genotype	*N*	% Rhythmic	Period Mean ± SEM
*w*^1118^;+/GD3018 (*puc*)	13	100	23.57 ± 0.08
*w*^1118^;*tim*Gal4/GD3018	16	100	24.40 ± 0.05
*w*^1118^;GD 3018/+;*tim*Gal4/+	15	93	24.21 ± 0.16
*w*^1118^;GD3018/+;*pdf*Gal4/+	15	100	24.13 ± 0.06
*w*^1118^;+/GD3116 (*Ptp52F*)	13	92	23.92 ± 0.15
*w*^1118^;*tim*Gal4/GD3116	16	94	24.50 ± 0.09
*w*^1118^;GD3116/+;*tim*Gal4/+	9	78	24.36 ± 0.22
*w*^1118^;GD3116/+;*pdf*Gal4/+	8	75	23.92 ± 0.18
*w*^1118^;+/GD17123 (*TbCMF46*)	16	94	23.5
*w*^1118^;*tim*Gal4/GD17123	16	81	24.08 ± 0.14
*w*^1118^;GD17123/+;*tim*Gal4/+	15	100	24.00 ± 0.12
*w*^1118^;GD17123/+;*pdf*Gal4/+	16	88	23.89 ± 0.13
*w*^1118^;+/GD17760 (*stg*)	16	100	23.56 ± 0.04
*w*^1118^;*tim*Gal4/GD17760	16	94	24.10 ± 0.11
*w*^1118^;GD17760/+;*tim*Gal4/+	14	100	24.11 ± 0.14
*w*^1118^;GD17760/+;*pdf*Gal4/+	13	85	23.86 ± 0.15
*w*^1118^;+/GD19078 (CG17124)	16	100	23.50 ± 0.06
*w*^1118^;*tim*Gal4/GD19078	16	100	23.81 ± 0.10
*w*^1118^;GD19078/+;*tim*Gal4/+	16	88	24.36 ± 0.09
*w*^1118^;GD19078/+;*pdf*Gal4/+	12	100	24.04 ± 0.11
*w*^1118^;+/GD21611 (*CanB*)	16	100	23.03 ± 0.03
*w*^1118^;*tim*Gal4/GD21611	15	93	**24.64 ± 0.14**
*w*^1118^;GD21611/+;*tim*Gal4/+	14	100	24.14 ± 0.16
*w*^1118^;GD21611/+;*pdf*Gal4/+	16	100	23.66 ± 0.09
*w*^1118^;+/GD25317 (*Pp4-19C*)	14	100	23.71 ± 0.08
*w*^1118^;*tim*Gal4/GD25317	14	100	**24.93 ± 0.09**
*w*^1118^;GD25317/+;*tim*Gal4/+	15	93	24.39 ± 0.15
*w*^1118^;GD25317/+;*pdf*Gal4/+	16	100	23.72 ± 0.09
*w*^1118^;+/GD27232 (*Ptp4E*)	16	100	23.50 ± 0.08
*w*^1118^;*tim*Gal4/GD27232	16	100	24.41 ± 0.05
*w*^1118^;GD27232/+;*tim*Gal4/+	8	75	24.50 ± 0.20
*w*^1118^;GD27232/+;*pdf*Gal4/+	13	100	**24.54 ± 0.88**
*w*^1118^;+/GD32283 (*CanA1*)	16	100	23.50 ± 0.04
*w*^1118^;*tim*Gal4/GD32283	14	93	24.39 ± 0.23
*w*^1118^;GD32283/+;*tim*Gal4/+	16	94	24.53 ± 0.10
*w*^1118^;GD32283/+;*pdf*Gal4/+	14	93	24.00 ± 0.13
*w*^1118^;+/GD32956 (CG17598)	16	94	23.90 ± 0.10
*w*^1118^;*tim*Gal4/GD32956	15	93	24.54 ± 0.09
*w*^1118^;GD32956/+;*tim*Gal4/+	14	**43**	24.58 ± 0.08
*w*^1118^;GD32956/+;*pdf*Gal4/+	16	94	24.20 ± 0.08
*w*^1118^;+/GD35025 (*Pp1-87B*)	15	100	23.83 ± 0.10
*w*^1118^;*tim*Gal4/GD35025	16	100	24.63 ± 0.08
*w*^1118^;GD35025/+;*tim*Gal4/+	13	100	24.23 ± 0.12
*w*^1118^;GD35025/+;*pdf*Gal4/+	16	100	24.34 ± 0.12
*w*^1118^;+/GD41912 (*Ptpa*)	16	100	23.69 ± 0.09
*w*^1118^;*tim*Gal4/GD41912	15	100	24.57 ± 0.04
*w*^1118^;GD41912/+;*tim*Gal4/+	15	80	24.04 ± 0.15
*w*^1118^;GD41912/+;*pdf*Gal4/+	11	100	24.23 ± 0.16
*w*^1118^;+/GD41924 (*mts*)	16	100	23.53 ± 0.03
*w*^1118^;*tim*Gal4/GD41924	16	88	**25.14 ± 0.15**
*w*^1118^;GD41924/+;*tim*Gal4/+	11	100	**24.73 ± 0.08**
*w*^1118^;GD41924/+;*pdf*Gal4/+	14	93	**24.73 ± 0.08**
*w*^1118^;+/GD42051 (*sds22*)	14	86	23.71 ± 0.09
*w*^1118^;*tim*Gal4/GD42051	16	100	**24.81 ± 0.06**
*w*^1118^;GD42051/+;*tim*Gal4/+	16	94	**24.87 ± 0.11**
*w*^1118^;GD42051/+;*pdf*Gal4/+	14	100	24.14 ± 0.12
*w*^1118^;+/GD45415 (*Mkp3*)	15	93	23.78 ± 0.09
*w*^1118^;*tim*Gal4/GD45415	15	100	24.67 ± 0.08
*w*^1118^;GD45415/+;*tim*Gal4/+	15	80	24.33 ± 0.15
*w*^1118^;GD45415/+;*pdf*Gal4/+	14	93	24.38 ± 0.10
*w*^1118^;+/GD49671 (*Pp2a-29B*)	15	100	23.70 ± 0.06
*w*^1118^;*tim*Gal4/GD49671	30	—	—
*w*^1118^;GD49671/+;*tim*Gal4/+	15	100	**26.13 ± 0.09**
*w*^1118^;GD49671/+;*pdf*Gal4/+	16	94	**26.17 ± 0.20**
*w*^1118^;+/KK100121 (CG6380)	16	100	23.63 ± 0.07
*w*^1118^;*tim*Gal4/KK100121	14	**29**	23.38 ± 0.13
*w*^1118^;KK100121/+;*tim*Gal4/+	9	**0**	**AR**
*w*^1118^;KK100121/+;*pdf*Gal4/+	15	**7**	23.5
*w*^1118^;+/KK100178 (CG17746)	16	88	23.43 ± 0.05
*w*^1118^;*tim*Gal4/KK100178	12	**0**	**AR**
*w*^1118^;KK100178/+;*tim*Gal4/+	12	**33**	**25.88 ± 0.32**
*w*^1118^;KK100178/+;*pdf*Gal4/+	12	**0**	**AR**
*w*^1118^;+/KK100216 (CG2104)	16	94	23.5
*w*^1118^;*tim*Gal4/KK100216	16	63	23.55 ± 0.05
*w*^1118^;KK100216/+;*tim*Gal4/+	15	53	23.75 ± 0.15
*w*^1118^;KK100216/+;*pdf*Gal4/+	14	100	23.61 ± 0.06
*w*^1118^;+/KK100283 (*Mapmodulin*)	15	100	23.07 ± 0.06
*w*^1118^;*tim*Gal4/KK100283	16	81	23.89 ± 0.21
*w*^1118^;KK100283/+;*tim*Gal4/+	14	93	24.04 ± 0.18
*w*^1118^;KK100283/+;*pdf*Gal4/+	16	100	23.78 ± 0.11
*w*^1118^;+/KK100593 (*Gbs-70E*)	16	100	23.53 ± 0.03
*w*^1118^;*tim*Gal4/KK100593	13	54	**25.21 ± 0.33**
*w*^1118^;KK100593/+;*tim*Gal4/+	16	88	**25.46 ± 0.17**
*w*^1118^;KK100593/+;*pdf*Gal4/+	13	**8**	23.5
*w*^1118^;+/KK100914 (CG42327)	16	100	23.53 ± 0.03
*w*^1118^;*tim*Gal4/KK100914	16	100	23.78 ± 0.11
*w*^1118^;KK100914/+;*tim*Gal4/+	15	80	23.5
*w*^1118^;KK 100914/+;*pdf*Gal4/+	16	100	23.53 ± 0.03
*w*^1118^;+/KK101257 (*Ppm1*)	28	89	23.50 ± 0.06
*w*^1118^;*tim*Gal4/KK101257	15	**20**	**24.67 ± 0.44**
*w*^1118^;KK101257/+;*tim*Gal4/+	13	**23**	**24.67 ± 0.17**
*w*^1118^;KK101257/+;*pdf*Gal4/+	15	**40**	23.42 ± 0.08
*w*^1118^;+/KK101335 (CG10376)	16	100	23.5
*w*^1118^;*tim*Gal4/KK101335	16	94	24.30 ± 0.12
*w*^1118^;KK101335/+;*tim*Gal4/+	16	88	24.32 ± 0.53
*w*^1118^;KK101335/+;*pdf*Gal4/+	16	88	23.75 ± 0.17
*w*^1118^;+/KK101406 (*wdb*)	15	87	23.42 ± 0.05
*w*^1118^;*tim*Gal4/KK101406	16	88	24.20 ± 0.09
*w*^1118^;KK101406/+;*tim*Gal4/+	15	53	**25.86 ± 0.23**
*w*^1118^;KK101406/+;*pdf*Gal4/+	13	92	23.83 ± 0.09
*w*^1118^;+/KK101474 (*Clb2*)	16	100	23.69 ± 0.09
*w*^1118^;*tim*Gal4/KK101474	16	88	23.43 ± 0.16
*w*^1118^;KK101474/+;*tim*Gal4/+	16	88	23.61 ± 0.14
*w*^1118^;KK101474/+;*pdf*Gal4/+	14	100	23.57 ± 0.05
*w*^1118^;+/KK101547 (*I-2*)	16	94	23.47 ± 0.03
*w*^1118^;*tim*Gal4/KK101547	14	**43**	**25.25 ± 0.35**
*w*^1118^;KK101547/+;*tim*Gal4/+	16	**31**	**25.40 ± 0.17**
*w*^1118^;KK101547/+;*pdf*Gal4/+	13	**23**	24.83 ± 0.59
*w*^1118^;+/KK101997 (*PpV*)	16	100	23.44 ± 0.04
*w*^1118^;*tim*Gal4/KK101997	12	50	23.50 ± 0.20
*w*^1118^;KK101997/+;*tim*Gal4/+	12	67	**24.81 ± 0.34**
*w*^1118^;KK101997/+;*pdf*Gal4/+	10	**0**	**AR**
*w*^1118^;+/KK102021 (*PpY-55A*)	16	100	23.47 ± 0.03
*w*^1118^;*tim*Gal4/KK102021	15	93	24.21 ± 0.17
*w*^1118^;KK102021/+;*tim*Gal4/+	14	64	23.78 ± 0.18
*w*^1118^;KK102021/+;*pdf*Gal4/+	14	100	23.89 ± 0.18
*w*^1118^;+/KK102060 (*PpN-58A*)	28	96	23.50 ± 0.03
*w*^1118^;*tim*Gal4/KK102060	15	60	24.06 ± 0.18
*w*^1118^;KK102060/+;*tim*Gal4/+	15	60	24.39 ± 0.14
*w*^1118^;KK102060/+;*pdf*Gal4/+	13	92	23.92 ± 0.18
*w*^1118^;+/KK102071 (CG14297)	25	100	23.53 ± 0.05
*w*^1118^;*tim*Gal4/KK102071	15	67	23.55 ± 0.17
*w*^1118^;KK102071/+;*tim*Gal4/+	16	75	24.00 ± 0.19
*w*^1118^;KK102071/+;*pdf*Gal4/+	16	94	23.50 ± 0.05
*w*^1118^;+/KK102397 (*Ptp36E*)	16	100	23.63 ± 0.07
*w*^1118^;*tim*Gal4/KK102397	14	100	24.21 ± 0.11
*w*^1118^;KK102397/+;*tim*Gal4/+	15	93	24.12 ± 0.14
*w*^1118^;KK102397/+;*pdf*Gal4/+	15	80	23.54 ± 0.09
*w*^1118^;+/KK102474 (CG31469)	15	100	23.73 ± 0.08
*w*^1118^;*tim*Gal4/KK102474	16	100	24.59 ± 0.12
*w*^1118^;KK102474/+;*tim*Gal4/+	14	93	23.73 ± 0.09
*w*^1118^;KK102474/+;*pdf*Gal4/+	16	94	23.77 ± 0.08
*w*^1118^;+/KK103044 (*Gbs-76A*)	16	81	23.38 ± 0.06
*w*^1118^;*tim*Gal4/KK103044	16	75	23.71 ± 0.11
*w*^1118^;KK103044/+;*tim*Gal4/+	15	67	**24.85 ± 0.10**
*w*^1118^;KK103044/+;*pdf*Gal4/+	14	100	23.93 ± 0.07
*w*^1118^;+/KK103144 (*Pp2B-14D*)	16	94	23.47 ± 0.06
*w*^1118^;*tim*Gal4/KK103144	16	56	24.11 ± 0.27
*w*^1118^;KK103144/+;*tim*Gal4/+	10	70	24.21 ± 0.32
*w*^1118^;KK103144/+;*pdf*Gal4/+	13	92	23.88 ± 0.13
*w*^1118^;+/KK103317 (CG32568)	16	100	23.53 ± 0.03
*w*^1118^;*tim*Gal4/KK103317	16	94	23.83 ± 0.12
*w*^1118^;KK103317/+;*tim*Gal4/+	12	100	23.96 ± 0.12
*w*^1118^;KK103317/+;*pdf*Gal4/+	16	100	23.53 ± 0.05
*w*^1118^;+/KK103354 (CG7115)	15	100	23.6 ± 0.05
*w*^1118^;*tim*Gal4/KK103354	14	86	**24.88 ± 0.2**
*w*^1118^;KK103354/+;*tim*Gal4/+	7	54	**25.0 ± 0.18**
*w*^1118^;KK103354/+;*pdf*Gal4/+	12	**0**	**AR**
*w*^1118^;+/KK103357 (*Cep97*)	15	100	23.57 ± 0.08
*w*^1118^;*tim*Gal4/KK103357	12	**25**	23.83 ± 0.27
*w*^1118^;KK103357/+;*tim*Gal4/+	13	**8**	**25.0**
*w*^1118^;KK103357/+;*pdf*Gal4/+	10	**0**	**AR**
*w*^1118^;+/KK103627 (*cdc14*)	16	100	23.34 ± 0.06
*w*^1118^;*tim*Gal4/KK103627	15	73	24.05 ± 0.22
*w*^1118^;KK103627/+;*tim*Gal4/+	15	93	**25.32 ± 0.14**
*w*^1118^;KK103627/+;*pdf*Gal4/+	13	**8**	**26.5**
*w*^1118^;+/KK103740 (*Ptpmeg*)	16	94	23.40 ± 0.05
*w*^1118^;*tim*Gal4/KK103740	16	88	23.54 ± 0.03
*w*^1118^;KK103740/+;*tim*Gal4/+	15	87	23.77 ± 0.14
*w*^1118^;KK103740/+;*pdf*Gal4/+	16	100	23.5
*w*^1118^;+/KK104081 (CG32812)	11	100	23.5
*w*^1118^;*tim*Gal4/KK104081	16	100	23.63 ± 0.07
*w*^1118^;KK104081/+;*tim*Gal4/+	16	88	23.61 ± 0.08
*w*^1118^;KK104081/+;*pdf*Gal4/+	16	94	23.73 ± 0.09
*w*^1118^;+/KK104167 (*tws*)	13	54	23.14 ± 0.09
*w*^1118^;*tim*Gal4/KK104167	15	87	23.85 ± 0.14
*w*^1118^;KK104167/+;*tim*Gal4/+	12	75	23.89 ± 0.20
*w*^1118^;KK104167/+;*pdf*Gal4/+	16	94	23.80 ± 0.13
*w*^1118^;+/KK104211 (*PpD6*)	15	93	23.42 ± 0.04
*w*^1118^;*tim*Gal4/KK104211	14	**29**	**24.88 ± 0.52**
*w*^1118^;KK104211/+;*tim*Gal4/+	12	58	**25.14 ± 0.37**
*w*^1118^;KK104211/+;*pdf*Gal4/+	12	**8**	**25.5**
*w*^1118^;+/KK104374 (*Mkp*)	14	86	23.5
*w*^1118^;*tim*Gal4/KK104374	16	88	23.46 ± 0.04
*w*^1118^;KK104374/+;*tim*Gal4/+	14	93	24.00 ± 0.17
*w*^1118^;KK104374/+;*pdf*Gal4/+	15	100	24.03 ± 0.06
*w*^1118^;+/KK104427 (*Ptpmeg2*)	16	94	23.57 ± 0.12
*w*^1118^;*tim*Gal4/KK104427	13	**0**	**AR**
*w*^1118^;KK104427/+;*tim*Gal4/+	13	54	**25.00 ± 0.68**
*w*^1118^;KK104427/+;*pdf*Gal4/+	10	**10**	23.5
*w*^1118^;+/KK104452 (*PpD5*)	16	100	23.50 ± 0.04
*w*^1118^;*tim*Gal4/KK104452	16	**44**	**25.29 ± 0.33**
*w*^1118^;KK104452/+;*tim*Gal4/+	15	67	24.35 ± 0.21
*w*^1118^;KK104452/+;*pdf*Gal4/+	13	**15**	23.25 ± 0.18
*w*^1118^;+/KK104677 (*flw*)	15	100	23.5
*w*^1118^;*tim*Gal4/KK104677	16	94	24.5
*w*^1118^;KK104677/+;*tim*Gal4/+	16	100	23.97 ± 0.13
*w*^1118^;KK104677/+;*pdf*Gal4/+	16	100	24.31 ± 0.08
*w*^1118^;+/KK104729 (CG11597)	16	88	23.29 ± 0.07
*w*^1118^;*tim*Gal4/KK104729	13	69	23.61 ± 0.07
*w*^1118^;KK104729/+;*tim*Gal4/+	11	91	23.70 ± 0.08
*w*^1118^;KK104729/+;*pdf*Gal4/+	16	94	23.80 ± 0.12
*w*^1118^;+/KK104761 (*Ptp69D*)	15	100	23.53 ± 0.03
*w*^1118^;*tim*Gal4/KK104761	12	**17**	**24.75 ± 0.18**
*w*^1118^;KK104761/+;*tim*Gal4/+	13	54	**25.43 ± 0.34**
*w*^1118^;KK104761/+;*pdf*Gal4/+	8	**0**	**AR**
*w*^1118^;+/KK104774 (*PTPMT1*)	15	100	23.47 ± 0.03
*w*^1118^;*tim*Gal4/KK104774	15	93	24.18 ± 0.14
*w*^1118^;KK104774/+;*tim*Gal4/+	14	86	**24.63 ± 0.24**
*w*^1118^;KK104774/+;*pdf*Gal4/+	8	88	23.36 ± 0.09
*w*^1118^;+/KK104785 (*Dd*)	16	100	23.03 ± 0.03
*w*^1118^;*tim*Gal4/KK104785	16	100	23.94 ± 0.13
*w*^1118^;KK104785/+;*tim*Gal4/+	16	100	23.97 ± 0.11
*w*^1118^;KK104785/+;*pdf*Gal4/+	16	94	23.70 ± 0.15
*w*^1118^;+/KK104860 (*mop*)	16	100	23.5
*w*^1118^;*tim*Gal4/KK104860	13	92	24.25 ± 0.14
*w*^1118^;KK104860/+;*tim*Gal4/+	15	80	**24.83 ± 0.31**
*w*^1118^;KK104860/+;*pdf*Gal4/+	16	81	23.62 ± 0.11
*w*^1118^;+/KK104884 (*MKP-4*)	16	90	23.37 ± 0.04
*w*^1118^;*tim*Gal4/KK104884	15	**33**	**24.40 ± 0.40**
*w*^1118^;KK104884/+;*tim*Gal4/+	12	67	**25.44 ± 0.18**
*w*^1118^;KK104884/+;*pdf*Gal4/+	11	**27**	24.00 ± 0.41
*w*^1118^;+/KK105122 (CG13197)	16	88	23.46 ± 0.03
*w*^1118^;*tim*Gal4/KK105122	16	94	23.83 ± 0.12
*w*^1118^;KK105122/+;*tim*Gal4/+	16	100	23.69 ± 0.12
*w*^1118^;KK105122/+;*pdf*Gal4/+	15	100	24.07 ± 0.16
*w*^1118^;+/KK105249 (*Pp2C*)	16	100	23.33 ± 0.04
*w*^1118^;*tim*Gal4/KK105249	16	**31**	24.20 ± 0.30
*w*^1118^;KK105249/+;*tim*Gal4/+	13	62	**25.56 ± 0.20**
*w*^1118^;KK105249/+;*pdf*Gal4/+	16	100	23.78 ± 0.08
*w*^1118^;+/KK105399 (*PPP4R2r*)	13	100	23.65 ± 0.05
*w*^1118^;*tim*Gal4/KK105399	12	**25**	23.17 ± 0.17
*w*^1118^;KK105399/+;*tim*Gal4/+	7	**0**	**AR**
*w*^1118^;KK105399/+;*pdf*Gal4/+	9	78	23.00 ± 0.25
*w*^1118^;+/KK105483 (*alph*)	16	100	23.5
*w*^1118^;*tim*Gal4/KK105483	16	88	23.89 ± 0.10
*w*^1118^;KK105483/+;*tim*Gal4/+	16	94	23.73 ± 0.13
*w*^1118^;KK105483/+;*pdf*Gal4/+	13	100	24.50 ± 0.13
*w*^1118^;+/KK105484 (CG15528)	13	77	23.25 ± 0.08
*w*^1118^;*tim*Gal4/KK105484	16	50	23.69 ± 0.26
*w*^1118^;KK105484/+;*tim*Gal4/+	14	**29**	23.63 ± 0.27
*w*^1118^;KK105484/+;*pdf*Gal4/+	15	100	23.90 ± 0.05
*w*^1118^;+/KK105525 (*Pp1*α*-96A*)	16	100	23.57 ± 0.05
*w*^1118^;*tim*Gal4/KK105525	10	50	**25.20 ± 0.34**
*w*^1118^;KK105525/+;*tim*Gal4/+	13	85	**25.50 ± 0.13**
*w*^1118^;KK105525/+;*pdf*Gal4/+	16	94	**24.73 ± 0.12**
*w*^1118^;+/KK105565 (*I-t*)	14	100	23.5
*w*^1118^;*tim*Gal4/KK105565	15	53	23.63 ± 0.08
*w*^1118^;KK105565/+;*tim*Gal4/+	16	56	23.56 ± 0.05
*w*^1118^;KK105565/+;*pdf*Gal4/+	11	55	23.47 ± 0.06
*w*^1118^;+/KK105568 (CG6036)	16	100	23.5
*w*^1118^;*tim*Gal4/KK105568	16	56	24.28 ± 0.12
*w*^1118^;KK105568/+;*tim*Gal4/+	14	71	23.90 ± 0.19
*w*^1118^;KK105568/+;*pdf*Gal4/+	12	92	23.68 ± 0.10
*w*^1118^;+/KK105674 (CG5026)	16	100	23.47 ± 0.03
*w*^1118^;*tim*Gal4/KK105674	16	100	24.19 ± 0.12
*w*^1118^;KK105674/+;*tim*Gal4/+	16	100	23.97 ± 0.13
*w*^1118^;KK105674/+;*pdf*Gal4/+	16	100	23.5
*w*^1118^;+/KK106098 (CG7378)	16	100	23.53 ± 0.03
*w*^1118^;*tim*Gal4/KK106098	16	69	24.14 ± 0.27
*w*^1118^;KK106098/+;*tim*Gal4/+	16	94	**25.07 ± 0.15**
*w*^1118^;KK106098/+;*pdf*Gal4/+	13	54	24.00 ± 0.49
*w*^1118^;+/KK106180 (CG10417)	16	94	23.50 ± 0.05
*w*^1118^;*tim*Gal4/KK106180	14	**14**	**24.75 ± 0.18**
*w*^1118^;KK106180/+;*tim*Gal4/+	15	73	**24.64 ± 0.27**
*w*^1118^;KK106180/+;*pdf*Gal4/+	16	**13**	23.25 ± 0.18
*w*^1118^;+/KK106253 (*Fcp1*)	16	100	23.53 ± 0.03
*w*^1118^;*tim*Gal4/KK106253	11	100	24.36 ± 0.15
*w*^1118^;KK106253/+;*tim*Gal4/+	4	100	23.75 ± 0.22
*w*^1118^;KK106253/+;*pdf*Gal4/+	16	100	23.47 ± 0.03
*w*^1118^;+/KK107057 (*PP2A-B*’)	31	87	23.48 ± 0.06
*w*^1118^;*tim*Gal4/KK107057	16	50	**22.25 ± 0.34**
*w*^1118^;KK107057/+;*tim*Gal4/+	12	**42**	**22.30 ± 0.34**
*w*^1118^;KK107057/+;*pdf*Gal4/+	16	81	23.65 ± 0.14
*w*^1118^;+/KK107386 (*PpD3*)	8	100	23.5
*w*^1118^;*tim*Gal4/KK107386	12	100	24.04 ± 0.12
*w*^1118^;KK107386/+;*tim*Gal4/+	11	100	23.68 ± 0.16
*w*^1118^;KK107386/+;*pdf*Gal4/+	15	100	23.57 ± 0.04
*w*^1118^;+/KK107621 (CG4733)	16	100	23.56 ± 0.04
*w*^1118^;*tim*Gal4/KK107621	16	81	23.65 ± 0.09
*w*^1118^;KK107621/+;*tim*Gal4/+	16	100	23.66 ± 0.07
*w*^1118^;KK107621/+;*pdf*Gal4/+	15	100	23.63 ± 0.10
*w*^1118^;+/KK107770 (*Pp1-^13^C*)	15	100	23.43 ± 0.04
*w*^1118^;*tim*Gal4/KK107770	16	100	23.44 ± 0.08
*w*^1118^;KK107770/+;*tim*Gal4/+	16	94	23.63 ± 0.07
*w*^1118^;KK107770/+;*pdf*Gal4/+	15	93	23.43 ± 0.05
*w*^1118^;+/KK107996 (*Lar*)	16	100	23.53 ± 0.03
*w*^1118^;*tim*Gal4/KK107996	12	**25**	24.17 ± 0.27
*w*^1118^;KK107996/+;*tim*Gal4/+	16	**0**	**AR**
*w*^1118^;KK107996/+;*pdf*Gal4/+	13	**0**	**AR**
*w*^1118^;+/KK107998 (*ssh*)	16	100	23.5
*w*^1118^;*tim*Gal4/KK107998	15	93	24.07 ± 0.10
*w*^1118^;KK107998/+;*tim*Gal4/+	15	100	24.30 ± 0.15
*w*^1118^;KK107998/+;*pdf*Gal4/+	16	100	23.44 ± 0.04
*w*^1118^;+/KK108071 (*eya*)	16	100	23.5
*w*^1118^;*tim*Gal4/KK108071	16	88	24.11 ± 0.13
*w*^1118^;KK108071/+;*tim*Gal4/+	16	100	23.66 ± 0.09
*w*^1118^;KK108071/+;*pdf*Gal4/+	16	100	23.5
*w*^1118^;+/KK108352 (*csw*)	15	100	23.60 ± 0.05
*w*^1118^;*tim*Gal4/KK108352	16	100	24.41 ± 0.08
*w*^1118^;KK108352/+;*tim*Gal4/+	16	94	24.23 ± 0.09
*w*^1118^;KK108352/+;*pdf*Gal4/+	15	87	23.62 ± 0.08
*w*^1118^;+/KK108505 (*Ptp99A*)	16	100	23.5
*w*^1118^;*tim*Gal4/KK108505	13	92	23.71 ± 0.11
*w*^1118^;KK108505/+;*tim*Gal4/+	16	100	23.56 ± 0.04
*w*^1118^;KK108505/+;*pdf*Gal4/+	16	100	23.75 ± 0.09
*w*^1118^;+/KK108744 (CG10089)	15	87	23.38 ± 0.10
*w*^1118^;*tim*Gal4/KK108744	14	86	24.00 ± 0.12
*w*^1118^;KK108744/+;*tim*Gal4/+	15	**47**	24.29 ± 0.14
*w*^1118^;KK108744/+;*pdf*Gal4/+	16	81	23.73 ± 0.14
*w*^1118^;+/KK108802 (CG8509)	14	100	23.54 ± 0.03
*w*^1118^;*tim*Gal4/KK108802	14	93	24.00 ± 0.09
*w*^1118^;KK108802/+;*tim*Gal4/+	16	81	24.15 ± 0.14
*w*^1118^;KK108802/+;*pdf*Gal4/+	15	93	23.79 ± 0.07
*w*^1118^;+/KK108859 (*NIPP1*)	15	80	23.46 ± 0.09
*w*^1118^;*tim*Gal4/KK108859	16	75	23.86 ± 0.13
*w*^1118^;KK108859/+;*tim*Gal4/+	12	83	**24.71 ± 0.23**
*w*^1118^;KK108859/+;*pdf*Gal4/+	15	100	23.80 ± 0.09
*w*^1118^;+/KK108888 (*Ptp61F*)	16	100	23.72 ± 0.08
*w*^1118^;*tim*Gal4/KK108888	16	94	23.70 ± 0.14
*w*^1118^;KK108888/+;*tim*Gal4/+	16	94	23.67 ± 0.09
*w*^1118^;KK108888/+;*pdf*Gal4/+	16	94	23.70 ± 0.10
*w*^1118^;+/KK109147 (*PP1-Y2*)	16	100	23.75 ± 0.06
*w*^1118^;*tim*Gal4/KK109147	14	**14**	23.5
*w*^1118^;KK109147/+;*tim*Gal4/+	11	**18**	**24.75 ± 0.18**
*w*^1118^;KK109147/+;*pdf*Gal4/+	12	67	**25.06 ± 0.78**
*w*^1118^;+/KK109622 (CG14411)	16	94	23.53 ± 0.03
*w*^1118^;*tim*Gal4/KK109622	16	100	**24.50 ± 0.13**
*w*^1118^;KK109622/+;*tim*Gal4/+	12	75	24.06 ± 0.17
*w*^1118^;KK109622/+;*pdf*Gal4/+	16	94	23.60 ± 0.10
*w*^1118^;+/KK109858 (*CanA-14F*)	16	100	23.5
*w*^1118^;*tim*Gal4/KK109858	11	**45**	24.08 ± 0.27
*w*^1118^;KK109858/+;*tim*Gal4/+	10	**10**	23.5
*w*^1118^;KK109858/+;*pdf*Gal4/+	11	**0**	**AR**
*w*^1118^;+/KK110167 (CG3632)	16	100	23.5
*w*^1118^;*tim*Gal4/KK110167	16	94	23.70 ± 0.10
*w*^1118^;KK110167/+;*tim*Gal4/+	15	87	23.73 ± 0.21
*w*^1118^;KK110167/+;*pdf*Gal4/+	13	85	23.45 ± 0.04
*w*^1118^;+/KK110360 (*Phlpp*)	16	88	23.40 ± 0.07
*w*^1118^;*tim*Gal4/KK110360	14	93	24.00 ± 0.08
*w*^1118^;KK110360/+;*tim*Gal4/+	16	100	23.75 ± 0.10
*w*^1118^;KK110360/+;*pdf*Gal4/+	16	81	23.81 ± 0.27
*w*^1118^;+/KK110443 (*Ptp10D*)	16	100	23.59 ± 0.05
*w*^1118^;*tim*Gal4/KK110443	15	73	24.05 ± 0.25
*w*^1118^;KK110443/+;*tim*Gal4/+	13	69	**25.28 ± 0.26**
*w*^1118^;KK110443/+;*pdf*Gal4/+	14	71	23.55 ± 0.22
*w*^1118^;+/KK110595 (*IA-2*)	16	100	23.72 ± 0.12
*w*^1118^;*tim*Gal4/KK110595	12	67	24.42 ± 0.08
*w*^1118^;KK110595/+;*tim*Gal4/+	16	63	24.70 ± 0.08
*w*^1118^;KK110595/+;*pdf*Gal4/+	5	80	24.50 ± 0.31
*w*^1118^;+/KK110786 (CG3530)	16	100	23.5
*w*^1118^;*tim*Gal4/KK110786	16	88	**25.36 ± 0.08**
*w*^1118^;KK110786/+;*tim*Gal4/+	16	94	**25.37 ± 0.11**
*w*^1118^;KK110786/+;*pdf*Gal4/+	14	**7**	**27.0**

Adult males were entrained in LD for 3 d and transferred to DD for at least 7 d. Analysis of activity rhythms in DD and fly genotypes are as described in *Methods and Materials*. For each RNAi line tested, the gene name or CG number is listed in parenthesis after the control RNAi only genotype. *N*, number of animals tested; % Rhythmic, percentage of rhythmic animals; Period ± SEM, rhythm period in hours ± SEM. Genotypes that lack SEM values all fell into the same half hour period increment. Bold “% Rhythmic” values signify <50% rhythmicity, bold “Period ± SEM” values are significantly different (*p* ≤ 0.05) from their respective UAS-RNAi/+ control flies, and “—” indicates that flies of the given genotype did not survive the run of the assay. AR, arrhythmic; LD, 12h:12h light-dark cycle; DD, complete darkness; RNAi, RNA interference.

Based on these criteria, candidate phosphatases that mediate *Drosophila* circadian clock function include: *mts* (GD41924), *Pp2A-29B* (GD49671), CG6380 (KK100121), CG17746 (KK100178), *Gbs-70E* (KK100593), *Ppm1* (KK101257), *I-2* (KK101547), CG7115 (KK103354), *Cep97* (KK103357), *PpD6* (KK104211), *Ptpmeg2* (KK104427), *Ptp69D* (KK104761), *MKP-4* (KK104884), *Pp1*α*-96A* (KK105525), CG10417 (KK106180), *Lar* (KK107996), *Pp1-Y2* (KK109147), *CanA-14F* (KK109858), and CG3530 (KK110786). Next, the efficacy and specificity of RNAi-mediated knockdown of these candidate “clock phosphatases” was validated by testing RNAi lines that target another region of the mRNA and/or other genetic reagents (*i.e.*, transposon inserts, Gal4/UAS system driven overexpression, and existing loss-of-function mutants) for activity rhythm defects. For each candidate clock phosphatase, we provide a description of the protein, its known functions, and the results of the genetic reagents used to verify the RNAi phenotype below.

### Microtubule star (mts)

MTS is the catalytic subunit of a protein phosphatase 2a (Pp2A) that dephosphorylates proteins at serine and threonine residues. It functions in many cellular process including the mitotic cell cycle, cell surface receptor signaling, and cell adhesion (Janssens and Goris 2001; Chen *et al.* 2007). Importantly, previous work demonstrated that MTS modulates PER nuclear localization and CLK transcriptional activity within the *Drosophila* circadian clock (Sathyanarayanan *et al.* 2004; Andreazza *et al.* 2015). Behavioral analysis of an additional RNAi line that targeted another region of the mRNA did not validate the initial screen phenotype, emphasizing the importance of validating RNAi phenotypes using independent genetic reagents. In this case, overexpressing *mts* (UAS-*mts*) using clock cell-specific Gal4 drivers disrupted activity rhythms, thus independently validating the RNAi screen results (Table 2).

**Table 2:** Activity rhythms of strains used to validate candidate clock phosphatases

Genotype	*n*	% Rhythmic	Period Mean ± SEM
*w*^1118^;+/GD35171	43	100	23.73 ± 0.04
*w*^1118^;*tim*Gal4/GD35171	16	94	24.43 ± 0.08
*w*^1118^;GD35171/+;*tim*Gal4/+	11	91	24.50 ± 0.19
*w*^1118^;GD35171/+;*pdf*Gal4/+	15	93	23.82 ± 0.07
*w*^1118^;;UAS-*mts*/+	12	100	23.5
*w*^1118^;*tim*Gal4/+;UAS-*mts*/+	8	**0**	**AR**
*w*^1118^;;UAS-*mts*/*pdf*Gal4	14	**43**	23.30 ± 0.18
*w*^1118^;P{EP}*Pp2A-29B*^EP2332^/+	16	100	23.63 ± 0.05
*w*^1118^;P{EP}*Pp2A-29B*^EP2332^/*tim*Gal4	15	100	23.90 ± 0.13
*w*^1118^;P{RS3}*Pp2A-29B*^CB-5426-3^	16	88	23.57 ± 0.05
*w*^1118^;PBac{WH}CG17746^f05041^	11	91	23.30 ± 0.08
y^1^w*;P{EP}CG17746^G4827^	16	94	23.53 ± 0.06
*y*^1^*w*^1118^;PBac{3HPy^+^}*I-2*^C362^	14	100	23.75 ± 0.13
w*;P{UAS-*I-2*.HA}G/+;P{UAS-Pp1-87B.HA}^1^H/+	15	100	23.67 ± 0.11
w*;P{UAS*-I-2*.HA}G/*tim*Gal4;P{UAS-Pp1-87B.HA}^1^H/+	15	87	24.15 ± 0.15
w*;P{UAS-*I-2*.HA}G/+;P{UAS-Pp1-87B.HA}^1^H/*pdf*Gal4	9	100	24.22 ± 0.11
y^1^w^67c23^;P{SUPor-P}CG7115^KG02655^	12	75	23.50 ± 0.14
*w*^1118^;UAS-*Cep97*/+	15	100	24.27 ± 0.14
*w*^1118^;UAS-*Cep97*/*tim*Gal4	9	100	24.28 ± 0.08
*w*^1118^;UAS-*Cep97*/+;*pdf*Gal4/+	13	100	24.67 ± 0.54
UAS-*Cep97*/Y	14	93	23.38 ± 0.06
UAS-*Cep97*/Y;*tim*Gal4/+	15	60	23.83 ± 0.11
UAS-*Cep97*/Y;;*pdf*Gal4/+	13	85	23.59 ± 0.06
*w*^1118^;;UAS-*Cep97*/+	15	100	23.5
*w*^1118^;*tim*Gal4/+;UAS-*Cep97*/+	16	100	23.97 ± 0.10
*w*^1118^;;UAS-*Cep97*/*pdf*Gal4	16	100	24.31 ± 0.10
*y*^1^;P{SUPor-P}tocKG08989*PpD6*^KG08989^	11	100	23.68 ± 0.12
*y*^1^w*Mi{MIC}*Ptpmeg2*^MI03011^/Y	15	100	23.73 ± 0.08
w^67c23^P{lacW}*Ptpmeg2*^G0232^/Y	16	63	24.65 ± 0.21
*y*^1^*w*^67c23^P{Mae-UAS.6.11}*Ptpmeg2*^GG01129^/Y	16	94	23.57 ± 0.07
*y*^1^*w*^67c23^P{Mae-UAS.6.11}*Ptpmeg2*^GG01129^/Y;*tim*Gal4/+	10	90	24.22 ± 0.19
*y*^1^*w*^67c23^P{Mae-UAS.6.11}*Ptpmeg2*^GG01129^/Y;;*pdf*Gal4/+	15	100	23.80 ± 0.09
*w*^1118^PBac{WH}*Ptpmeg2*^f06600^/Y	13	100	23.62 ± 0.11
w*;;*Ptp69D*^1^	8	88	**26.57 ± 0.21**
w*;;Df(3L)8ex25	10	60	**26.5**
*w*^1118^;;*Ptp69D*^10^	15	93	**26.96 ± 0.14**
*w*^1118^;;*Ptp69D*^18^	16	50	**27.19 ± 0.15**
*w*^1118^;;*Ptp69D*^20^	10	100	23.60 ± 0.06
*w*^1118^;;*Ptp69D*^21^	16	100	23.53 ± 0.30
*w*^1118^;;UAS-*Ptp69D*/+	9	89	24.19 ± 0.22
*w*^1118^;;UAS-*Ptp69D/pdf*Gal4	12	67	24.13 ± 0.19
*w*^1118^;;*tim*Gal4/+;UAS-*Ptp69D*/+	12	92	**27.82 ± 0.28**
*w*^1118^;;UAS-DN*Ptp69D*/+	15	100	23.47 ± 0.09
*w*^1118^;;UAS-DN*Ptp69D*/*pdf*Gal4	8	63	**27.20 ± 0.18**
*w*^1118^;;*tim*Gal4/+;UAS-DN*Ptp69D*/+	15	100	23.50 ± 0.05
*w*^1118^;;*Ptp69D*^1^_iso_	8	100	23.44 ± 0.06
*y*^1^P{SUPor-P}MKP-4^KG03420^	13	100	24.23 ± 0.12
*w*^1118^;;P{GSV6}*Pp1*α*-96A*^GS11179^/+	16	100	23.59 ± 0.08
*w*^1118^;*tim*Gal4/+;P{GSV6}*Pp1*α*-96A*^GS11179^/+	16	**25**	24.13 ± 0.21
*w*^1118^;;P{GSV6}*Pp1*α*-96A*^GS11179^/*pdf*Gal4	9	67	23.67 ± 0.15
*w*^1118^;;*Pp1*α*-96A*^2^/+	16	94	23.77 ± 0.12
*w*^1118^;;UAS-*Pp1*α*-96A*.HA/+	16	100	23.38 ± 0.05
*w*^1118^;*tim*Gal4/+;UAS-*Pp1*α*-96A*.HA/+	16	94	23.87 ± 0.11
*w*^1118^;;UAS-*Pp1*α*-96A*.HA/*pdf*Gal4	16	94	23.33 ± 0.08
*w*^1118^;;*Pp1*α*-96A*-CRISPRmutant-1/+	17	82	23.52 ± 0.07
*w*^1118^;;*Pp1*α*-96A*-CRISPRmutant-2/+	13	92	23.58 ± 0.05
*w*^1118^;;*Pp1*α*-96A*-CRISPRmutant-3/+	9	100	23.44 ± 0.05
*w*^1118^;UAS-CG10417*/+*	16	100	23.44 ± 0.04
*w*^1118^;UAS-CG10417/*tim*Gal4	14	100	**24.96 ± 0.09**
*w*^1118^;UAS-CG10417/+;*pdf*Gal4/+	16	100	**24.97 ± 0.03**
w*;*Lar*^13.2^/+	14	93	23.54 ± 0.04
Df(2L)TW84,l(2)74i^1^,amos^Tft^*Lar*^TW84^/+	14	86	23.71 ± 0.11
Df(2L)E55,rdo^1^hook^1^*Lar*^E55^pr^1^/+	16	88	24.32 ± 0.08
*w*^1118^;;UAS-*Lar*/+	14	100	23.57 ± 0.04
*w*^1118^;*tim*Gal4/+;UAS-*Lar*/+	15	93	24.06 ± 0.07
*w*^1118^;;UAS-*Lar*/*pdf*Gal4	16	75	24.17 ± 0.07
*w*^1118^;*Lar*Df(2L)E55/*Lar*^13.2^;+	14	0	**AR**
*w*^1118^;UAS-*CanA-14F*myc/+	16	75	24.50 ± 0.20
*w*^1118^;UAS-*CanA-14F*myc/*tim*Gal4	16	63	23.60 ± 0.10
*w*^1118^;UAS-*CanA-14F*myc/+;*pdf*Gal4/+	15	100	24.03 ± 0.10
*w*^1118^;;UAS-*CanA-14F*act-myc/+	9	100	23.94 ± 0.11
*w*^1118^;*tim*Gal4/+;UAS-*CanA-14F*act-myc/+	15	**0**	**AR**
*w*^1118^;;UAS*-CanA-14F*act-myc/*pdf*Gal4	16	94	**25.03 ± 0.15**
*CanA-14F*-KO/Y	15	**13**	**25.0**
*CanA-14F*-KO_iso_/Y	16	94	23.67 ± 0.08
*w*^1118^;GD26216/+	12	100	23.63 ± 0.13
*w*^1118^;GD26216/*tim*Gal4	15	100	**24.90 ± 0.13**
*w*^1118^;GD26216/+;*tim*Gal4/+	13	85	23.68 ± 0.12
*w*^1118^;GD26216/+;*pdf*Gal4/+	15	100	24.30 ± 0.18

Adult males were entrained in LD for 3 d and transferred to DD for at least 7 d. Analysis of activity rhythms in DD and fly genotypes are as described in *Methods and Materials*. *n*, number of animals tested; % Rhythmic, percentage of rhythmic animals; Period ± SEM, rhythm period in hours ± SEM. Bold “% Rhythmic” values signify < 50% rhythmicity and bold “Period ± SEM” values are significantly different (*p* ≤ 0.05) from their respective UAS-RNAi/+ control flies. AR, arrhythmic; LD, 1h2:12h light-dark cycle; DD, complete darkness; RNAi, RNA interference.

### Pp2A-29B

Pp2A-29B is a protein phosphatase type 2A regulatory subunit. It functions in many cellular processes including chromosome segregation, centriole assembly, and phagocytosis (Stroschein-Stevenson *et al.* 2006; Dobbelaere *et al.* 2008). We tested additional *P* element transposon inserts to independently validate the RNAi phenotype, but activity rhythms were not altered (Table 2).

### CG6380

CG6380 is related to *Protein phosphatase inhibitor 2*, *IPP-2*. Based on association of InterPro records with GO terms, it functions to regulate signal transduction and phosphoprotein phosphatase pathways. Although the phenotype of this RNAi knockdown was strongly arrhythmic, no other genetic reagents were available to confirm the RNAi phenotype.

### CG17746

CG17746 is a member of the protein phosphatase 2C family that has cation binding domains and dephosphorylates proteins at serine and threonine residues. We tested additional *P* element transposon inserts for activity rhythms; however, these reagents did not validate the RNAi phenotype (Table 2).

### Gbs-70E

Gbs-70E is a protein phosphatase 1 regulatory subunit with a carbohydrate binding type-21 (CBM21) domain. It functions in regulation of glycogen metabolic process (Kerekes *et al.* 2014). However, there are no additional genetic reagents available for this gene to validate the arrhythmic RNAi phenotype.

### Ppm1

PPM1 is a protein phosphatase, Mg^+2^/Mn^+2^-dependent (PPM type), member of the protein phosphatase 2C family that dephosphorylates proteins at serine and threonine residues. There are no known cellular functions described for this phosphatase, and no additional genetic reagents available to validate the RNAi phenotype.

### I-2

I-2 is a protein phosphatase inhibitor with protein phosphatase 1 binding activity (Sami *et al.* 2011). Behavioral analysis of *P* element transposon insert and overexpression (UAS-*I-2*) driven with clock cell-specific Gal4 drivers did not reproduce a defect in activity rhythms consistent with the RNAi knockdown (Table 2).

### CG7115

CG7115 is a cation binding, PPM-type phosphatase, part of the protein phosphatase 2C family that dephosphorylates proteins at serine and threonine residues. It functions in many cellular processes including cell adhesion and regulation of cell shape (Sopko *et al.* 2014). We tested the available *P* element insert to independently validate the long period and/or arrhythmicity associated with RNAi, but did not observe any alteration in activity rhythms (Table 2).

### Cep97

Cep97 is a protein phosphatase type 1 regulator with a characteristic leucine-rich repeat domain. It is known to function in centriole replication (Dobbelaere *et al.* 2008). Behavioral analysis of *Cep97* overexpression using clock cell-specific Gal4 drivers did not alter activity rhythms (Table 2).

### PpD6

PpD6 is a protein phosphatase that dephosphorylates proteins at serine and threonine residues. There are no known cellular functions described for this phosphatase. We tested the only available *P* element transposon insert for this gene, but activity rhythms in this strain were not altered (Table 2).

### Ptpmeg2

Ptpmeg2, also known as *lethal-1-G0232*, is a nonmembrane spanning protein tyrosine phosphatase. It functions in many cellular processes including phagocytosis, neurogenesis, and cell migration (Stroschein-Stevenson *et al.* 2006; Chen *et al.* 2012). We tested the available *Ptpmeg2 P* element transposon insert lines and clock cell-specific *Ptpmeg2* overexpression flies, but none of these genetic reagents altered activity rhythms (Table 2).

### Ptp69D

Ptp69D is a protein tyrosine phosphatase with characteristic Fibronectin type III, Immunoglobulin subtype, and tyrosine-specific protein phosphatase domains. It is a transmembrane RPTP that dephosphorylates protein’s tyrosine residues. It functions in many cellular processes including dendrite morphogenesis, axon guidance, and fasciculation-defasciculation of neuron axons (Desai *et al.* 1996; Desai and Purdy 2003). Interestingly, when we tested additional *Ptp69D* reagents including loss-of-function mutants and a dominant negative UAS strain, many showed an even longer period phenotype compared to the RNAi while some showed no phenotype (Table 2). When *Ptp69D* mutants that showed a long period were isogenized to the wild-type (*w*^1118^) reference strain or paired with a wild-type X chromosome (data not shown), the long period phenotype was lost (Table 2). Upon further analysis (see *Methods and Materials*), we confirmed that the long period (∼26.5 hr) phenotype was due to *per^SLIH^*, a naturally occurring *per* mutant (Hamblen *et al.* 1998).

### MKP-4

MKP-4 is a dual specificity protein phosphatase that dephosphorylates proteins at tyrosine, serine, and threonine residues. Its known cellular function is in negative regulation of JUN kinase activity (Sun *et al.* 2008). We tested an additional *P* element transposon insert to independently validate the RNAi phenotype but activity rhythms were not altered (Table 2).

### Pp1α-96A

Pp1α-96A is a protein phosphatase, part of the PP1 subfamily, which dephosphorylates proteins at serine and threonine residues. It functions in many cellular processes including positive regulation of the canonical *Wnt* signaling pathway and innate immune response (Schertel *et al.* 2013). Importantly, the PP1 subfamily is proposed to regulate clock function in *Drosophila* by maintaining rhythms in PER-TIM abundance (Fang *et al.* 2007). None of the available *Pp1*α*-96A P* element inserts or clock cell-specific *Pp1*α*-96A* overexpression altered activity rhythms (Table 2). Given the involvement of PP1 in *Drosophila* circadian clocks, we wanted to test loss-of-function mutants. Therefore, we used CRISPR technology to generate three *Pp1*α*-96A* deletion mutants (See *Methods and Materials*). However, none of these mutants were homozygous viable as adults, and heterozygotes did not display altered activity rhythms (Table 2).

### CG10417

CG10417 is a PPM-type phosphatase, a member of the protein phosphatase 2C family that dephosphorylates proteins at the serine and threonine residues (Sopko *et al.* 2014). No other genetic reagents were available for this gene. However, given its association with the PP2 family known to be involved in *Drosophila* circadian clock function (Sathyanarayanan *et al.* 2004), we generated a UAS-CG10417 strain to overexpress this phosphatase in clock cells. Interestingly, CG10417 overexpression in clock cells resulted in a long period phenotype (Table 2). This phenotype is similar to that of the RNAi knockdown, demonstrating that increasing or decreasing the dephosphorylation of CG10417 targets slows the pace of the clock. This is not unprecedented, since RNAi knockdown and overexpression of the PP2A subunit WDB also leads to long period rhythms (Sathyanarayanan *et al.* 2004; Andreazza *et al.* 2015). Since CG10417 RNAi knockdown and overexpression both lengthened period, we investigated whether either manipulation altered CLK, PER, or TIM protein levels or phosphorylation, but no obvious change in either parameter was detected (data not shown).

### Lar

LAR is a transmembrane RPTP bearing Fibronectin type III, Immunoglobulin-like, and tyrosine-specific protein phosphatase domains. It is a transmembrane receptor protein tyrosine phosphatase that dephosphorylates proteins tyrosine residues (Streuli *et al.* 1989). It functions in many cellular processes including cell adhesion, axon guidance, and regulation of nervous system development (Krueger *et al.* 1996, 2003; Kiger *et al.* 2003). Although multiple loss-of-function mutants were available for this phosphatase, none were homozygous viable. However, one heterozygous combination of *Lar* loss-of-function alleles survived and phenocopied the arrhythmicity seen in *Lar* RNAi knockdown flies (Table 2). Further analysis showed that *Lar* is required for the development of circadian pacemaker neuron processes required for activity rhythms during constant darkness but not light:dark cycles (Agrawal and Hardin 2016).

### Pp1-Y2

Pp1-Y2 is a protein phosphatase that dephosphorylates proteins at the serine and threonine residues. The RNAi shows a strong arrhythmic and/or long period phenotype, but no additional genetic reagents were available to validate the RNAi phenotype.

### CanA-14F

CanA-14F is a protein phosphatase that dephosphorylates proteins at serine and threonine residues. It functions in many cellular processes including positive regulation of nuclear factor of activated T cells (NFAT) protein import into nucleus (Shibasaki *et al.* 1996) and sleep (Nakai *et al.* 2011). A *CanA-14F* knockout (KO) and a constitutively active *CanA-14F* form (expressed via Gal4/UAS) were generated previously (Nakai *et al.* 2011), and showed strong arrhythmic and long period activity phenotypes, respectively (Table 2). However, the arrhythmicity associated with *CanA-14F* KOs was lost when this allele was isogenized to a wild-type (*w*^1118^) reference strain (Table 2).

### CG3530

CG3530 is a protein phosphatase that dephosphorylates proteins at the tyrosine residues. Its known cellular function is in the mitotic cell cycle (Chen *et al.* 2007). Behavioral analysis of an additional RNAi line that targeted another region of the mRNA did not validate the initial screen phenotype (Table 2).

## Discussion

An *in vivo* screen of 86 RNAi lines, representing the majority of annotated *Drosophila* phosphatases/regulators, for altered activity rhythms was carried out. The screen identified a total of 19 candidate genes (Table 1) that altered clock function upon RNAi knockdown in *Drosophila* clock cells. Further genetic validation of one candidate showed that the RPTP *Lar* is required for the development of axonal projections from circadian pacemaker neurons that support rhythmic activity in constant darkness but not during light:dark cycles (Agrawal and Hardin 2016).

As expected, a majority of these candidates were not validated upon further analysis of independent genetic reagents (Table 2). However, these reagents consisted of additional *P* element inserts, where the *P* element insertion site may not interfere with gene function, or strains that could be used for overexpression, which also may not impact the function of a protein that is already at saturating levels. Therefore, a lack of validation with *P* element inserts and overexpression for these candidate clock phosphatases does not eliminate them from the list of viable candidates. However, for two candidate phosphatases, Ptp69D and CanA14F, loss-of-function mutants upon isogenization did not alter activity rhythms (Table 2), therefore these can be eliminated from the list of viable candidates.

Previous studies show that PP1 and PP2a both function within the *Drosophila* clock (Sathyanarayanan *et al.* 2004; Fang *et al.* 2007; Andreazza *et al.* 2015). PP1 is comprised of a catalytic subunit that engages with one of dozens of regulatory subunits to select substrates and control enzymatic activity (Peti *et al.* 2013). PP1 function in the *Drosophila* clock was assessed previously by overexpressing the nuclear inhibitor of PP1 (NIPP1), which reduced TIM levels and lengthened circadian period, indicating that PP1 dephosphorylates and stabilizes TIM to maintain circadian period (Fang *et al.* 2007). Since these circadian phenotypes were produced by generically inhibiting PP1, the clock-relevant catalytic and regulatory subunits involved were not identified. Five PP1 catalytic subunit genes (*flw*, *Pp1-^13^C*, *Pp1-87B*, *Pp1*α*-96A*, and *Pp1-Y2*) and eight PP1 regulator genes (*sds22*, *NIPP1*, *I-t*, *I-2*, TbCMF46, *Gbs-70A*, *Gbs-70E*, and *Cep97*) were tested in our screen, and *Pp1*α*-96A*, *Pp1-Y2*, *Gbs-70E*, *I-2*, and *Cep97* showed aberrant circadian phenotypes (Table 1). Further analysis of *Pp1*α*-96A* showed that none of the available *Pp1*α*-96A P* element inserts or overexpression of *Pp1*α*-96A* in clock cells altered activity rhythms (Table 2). Thus, we used the CRISPR/Cas9 system to generate three *Pp1*α*-96A* deletion mutants expected to disrupt Pp1α-96A protein expression and/or function (see *Methods and Materials*; Figure 1). However, none of these *Pp1*α*-96A* deletions were homozygous viable as adults, and heterozygotes did not display altered activity rhythms (Table 2). Further characterization of *Pp1*α*-96A* function in the clock will benefit from targeted loss of *Pp1*α*-96A* in clock cells. The *Pp1-Y2* catalytic subunit and the *Gbs-70E* and *I-2* regulators could not be tested further due to lack of genetic reagents and *Cep97* overexpression did not alter circadian rhythms (Table 2), but each of these genes remain as viable candidate clock protein phosphatases until additional loss-of-function reagents are available to test. Although inhibiting PP1 via NIPP1 overexpression lengthened circadian period (Sathyanarayanan *et al.* 2004), RNAi knockdown of NIPP1 did not alter activity rhythms (Table 1). These results suggest that NIPP1 RNAi is either ineffective, NIPP1 is not involved in suppressing PP1 activity in clock cells, or increased PP1 activity does not disrupt the circadian clock.

The PP2a holoenzyme contains a structural subunit Pp2A-29B, a catalytic subunit MTS, and regulatory subunits TWS, WDB, PP2a-B’, CG4733, and Connector of kinase to AP-1 (CKA) (Andreazza *et al.* 2015). Previous work shows that *mts*, *tws*, and *wdb* overexpression, hypomorphic mutants, and/or RNAi knockdown, alter the levels and localization of PER and disrupt activity rhythms (Sathyanarayanan *et al.* 2004; Andreazza *et al.* 2015), whereas a hypomorphic *cka* mutant and *cka* RNAi knockdown reduces CLK activity and lengthens period (Andreazza *et al.* 2015). We tested RNAi knockdowns of all PP2a components except *cka*, but only *mts* and *Pp2A-29B* produced circadian phenotypes (Table 1). Our inability to generate circadian phenotypes for *tws* and *wdb* may be due to inefficient RNAi knockdown, since expressing a different *wdb* RNAi along with *Dicer2* (to enhance RNAi potency) produced a long period phenotype (Andreazza *et al.* 2015). We did not test *cka* because it is not annotated as a PP2a subunit.

Genetic reagents for effecting a loss- or gain-of-function were not available for four additional candidate phosphatases, which can be characterized further when such reagents are available. For example, *P* element inserts are now available for *Gbs-70E* and CG3530. Another method that can be used to follow up on these candidates is to generate mutants using CRISPR technology. Since loss of most phosphatases is lethal, it is likely that generating conditional null mutants via CRISPR will provide the best opportunity to assess loss-of-function phenotypes in adults (Gratz *et al.* 2014).

Overall, we identified 19 protein phosphatases that may function within the *Drosophila* circadian clock (Table 3). *Lar* and *mts* functions have now been characterized, and they are shown to be important for dephosphorylation events that regulate fly clock development or function (Andreazza *et al.* 2015; Agrawal and Hardin 2016). *Ptp69D* and *CanA-14F* loss-of-function mutants do not alter activity rhythms upon isogenization, thus, identification of these genes may have been due to off-target effects of RNAi. No loss-of-function mutants are currently available for the remaining 15 candidates, and four of these candidates could not be validated due to the lack of independent genetic reagents (Table 3). We analyzed previous mRNA expression and CLK binding data that may further support a possible role for these candidates in the clock (Chintapalli *et al.* 2007; Celniker *et al.* 2009; Kula-Eversole *et al.* 2010; Abruzzi *et al.* 2011). Of the remaining 15 candidates, one is a rhythmic and two are nonrhythmic CLK binding targets, three are enriched in small LN_v_s, five are cycling in large LN_v_s, and five are highly or very highly expressed in pacemaker neuron-containing tissues (*i.e.*, brain and head) that could account for the altered activity rhythms due to candidate gene RNAi knockdown (Table 3). The other nine candidates were not detected as highly or very highly expressed transcripts in brains and heads, but moderate levels of transcripts were found in heads or brains for five of these candidates, leaving four candidates with low or no expression in the head (Table 3). One candidate (*i.e.*, *Ppm1*) that was detected in lLN_v_ pacemaker neurons showed low or no expression in the head (Table 3), consistent with there being only eight lLNvs per head (Helfrich-Forster 2014). The strong behavioral phenotypes displayed by RNAi knockdowns of these 15 candidates suggest they are viable candidate clock phosphatases, a possibility that is further supported by data showing that nine of these candidates are either CLK binding targets or produce cycling mRNAs in clock neurons (Table 3). Additional characterization of the remaining candidates may reveal novel features of the circadian timekeeping mechanism that are conserved in all animals including humans.

**Table 3:** Summary of results for candidate circadian phosphatases

Candidate	RNAi Phenotype	Genetic Reagents Tested	Validation	Spatial Expression*^a^*	Clock-Related Expression
*mts**^b^*	Long	Additional RNAi, overexpression	Yes	Brain, eye, tubule, carcass, ovary, heart, spermatheca, gut, fat body, head	CLK target and cycling*^c^*
***Pp2A-29B****^b^*	Long	*P* element inserts	No	Head, brain, tubule, ovary, testis, fat body, gut, salivary gland	CLK target and noncycling*^c^*
***IPP-2***	AR	No reagents	—	Testis	
**CG17746**	AR	*P* element inserts	No	Tubule, ovary	CLK target and noncycling*^c^*; cycling mRNA in large PDF neurons*^d^*
***Gbs-70E***	Long	No reagents	—	Head, carcass, ovary, heart, fat body, eye, crop, salivary gland, spermatheca	CLK target and cycling*^c^*; cycling mRNA in large PDF neurons*^d^*
***Ppm1***	AR	No reagents	—	Testis	Cycling mRNA in large PDF neurons*^d^*
***I-2***	AR	*P* element insert, overexpression	No	Head, tubule, carcass, ovary, testis, spermatheca, ganglion	
**CG7115**	Long	*P* element insert	No	Carcass, ovary	mRNA enriched in s-LN_v_s*^d^*
***Cep97***	AR	Overexpression strains	No	Testis	
***PpD6***	Long	*P* element insert	No	—	
***Ptpmeg2***	Long	*P* element inserts, overexpression	No	Head	Cycling mRNA in large PDF neurons*^d^*
*Ptp69D*	Long	Loss-of-function mutants, Dominant negative strain	No*^e^*	Ovary	mRNA enriched in s-LN_v_s; cycling mRNA in small and large PDF neurons*^d^*
***MKP-4***	Long	*P* element insert	No	—	Cycling mRNA in large PDF neurons*^d^*
***Pp1a-96A****^f^*	Long	*P* element inserts, Overexpression, CRISPR heterozygous mutants	No	Head, carcass, ovary, testis, crop, fat body, gut, salivary gland, spermatheca, accessory gland	mRNA enriched in s-LN_v_s*^d^*
**CG10417**	Long	Overexpression	Yes	Carcass, ovary	
*Lar*	AR	Deficiency over point mutant heterozygote	Yes	—	
***Pp1-Y2***	Long	No reagents	—	—	
*CanA-14F**^g^*	AR	Knockout, expression of constitutively active form	No*^h^*	Head, carcass, ovary	
**CG3530**	Long	Additional RNAi	No	Brain, head, ganglion	mRNA enriched in s-LN_v_s*^d^*

Bold denotes the remaining candidate clock protein phosphatases. RNAi, RNA interference; CLK, CLOCK; AR, arrhythmic; mRNA, messenger RNA; PDF, pigment dispersing factor; CRISPR, clustered regularly interspaced short palindromic repeats.

a High/very high expression level in adult fly tissues based on Celniker *et al.* (2009) and Chintapalli *et al.* (2007).

b Clock-related based on Sathyanarayanan *et al.* (2004).

c Direct CLK binding target and cycling or noncycling mRNA expression based on Abruzzi *et al.* (2011).

d Differential mRNA expression in pacemaker neurons based on Kula-Eversole *et al.* (2010).

e All genetic reagents used to verify the RNAi phenotype that produced long period rhythms were due to *per*^SLIH^.

f Clock-related based on Fang *et al.* (2007).

g Sleep-related based on previously analyzed for circadian phenotype Nakai *et al.* (2011).

h Knockout allele had a long period rhythm which was lost upon isogenization, but constitutively active form produces a long period rhythm upon overexpression.
